# Reduced expression of lamin A/C correlates with poor histological differentiation and prognosis in primary gastric carcinoma

**DOI:** 10.1186/1756-9966-28-8

**Published:** 2009-01-15

**Authors:** Zhengrong Wu, Lirong Wu, Desheng Weng, Dazhi Xu, Jian Geng, Fei Zhao

**Affiliations:** 1 Department of Pathology, Nanfang Hospital, Southern Medical University, Guangzhou, PR China; 2 Department of Pathology, School of Basic Medical Sciences, Southern Medical University, Guangzhou, PR China; 3 Guangdong Provincial Key Laboratory of Molecular Tumor Pathology, Guangzhou, PR China; 4 College of Traditional Chinese Medicine, Guangzhou University of Chinese Medicine, Guangzhou, PR China; 5 State Key Laboratory of Oncology in Southern China, the Cancer Center, Sun Yat-sen University, Guangzhou, PR China

## Abstract

**Background:**

Lamin A/C is very important in DNA replication, RNA dependent transcription and nuclear stabilization. Reduced or absent lamin A/C expression has been found to be a common feature of a variety of different cancers. To investigate the role of lamin A/C in gastric carcinoma (GC) pathogenesis, we analyzed the correlations between the lamin A/C expression level and clinicopathological factors and studied its prognostic role in primary GC.

**Methods:**

The expression of lamin A/C at mRNA level was detected by the reverse transcription-polymerase chain reaction (RT-PCR) and real time RT-PCR, and western blot was used to examine the protein expression. Lamin A/C expression and its prognostic significance were investigated by performing immunohistochemical analysis on a total of 126 GC clinical tissue samples.

**Results:**

Both lamin A/C mRNA and protein expression were downregulated in the majority of tumours compared with corresponding normal gastric tissues (*p *= 0.011 and *p *= 0.036, respectively). Real time RT-PCR further validated that downregulation of lamin A/C is associated with poor histological differentiation (r = 0.438, *p *= 0.025). The immunohistochemical staining showed an evident decrease of lamin A/C expression in 55.6% (70/126) GC cases. Importantly, the negative lamin A/C expression correlated strongly with histological classification (r = 0.361, *p *= 0.034). Survival analysis revealed that patients with lamin A/C downregulation have a poorer prognosis (*p *= 0.034). In addition, lamin A/C expression was found to be an independent prognostic factor by multivariate analysis.

**Conclusion:**

Data of this study suggest that lamin A/C is involved in the pathogenesis of GC, and it may serve as a valuable biomarker for assessing the prognosis for primary GC.

## Background

The A-type lamins (predominantly lamins A and C, two alternatively spliced products of the LMNA gene), along with B-type lamins (members of the intermediate filament proteins), are the most principal components of the nuclear lamina-a proteinaceous meshwork of 10 nm diameter filaments lying at the interface between chromatin and the inner nuclear membrane. The nuclear lamina is thought to be a principal determinant of nuclear architecture. Downregulation or specific mutations in lamins cause abnormal nuclear shape, changes in heterochromatin localization at the nuclear periphery, global chromatin reorganization and possibly specific changes in the positions of genes [1]. It is possible that changes in the nuclear lamina and in nuclear shape affect chromatin organization and gene positioning, respectively, and in this way alter patterns of gene expression, contributing to transformation [2].

Lamin A/C is important in DNA replication, chromatin anchoring, spatial orientation of nuclear pore complexes, RNA Pol II-dependent transcription and nuclear stabilization [3]. With regard to the multiple functions of A-type lamins, mutations in the human LMNA gene cause a wide range of heritable diseases collectively termed laminopathies [4].

Importantly, numerous studies suggest that reduced or absent lamin A/C expression is a common feature of a variety of different cancers, including small cell lung cancer (SCLC), skin basal cell and squamous cell carcinoma, testicular germ cell tumour, prostatic carcinoma, leukemia and lymphomas. The reduction in expression of lamin A/C frequently correlates with cancer subtypes and cancer aggressiveness, proliferative capacity and differentiation state [5].

Gastric cancer (GC) is the second most common cause of cancer-related death around the world [6] Although the number of death of patients undergoing surgical treatment for gastric cancer has decreased recently, GC is still a major health problem and a leading cause of cancer mortality in Asian countries. To identify reliable prognostic markers in GC is therefore very important to guide surgical and chemotherapeutic treatment. It had been reported that lamin A/C CpG island promoter hypermethylation is a significant predictor of shorter failure-free survival and overall survival in nodal diffuse large B-cell lymphomas. In addition, a series of experiments demonstrated that Lamin A/C is necessary for the retinoblastoma protein (pRB) stabilization and decreased expression of lamin A/C results in reduced activity of pRB. Hence, it is convincible to presume that change of lamin A/C protein may contribute to tumourigenesis and progression and may be a biomarker of malignancy. Moss et al [7] had reported that the expression of lamin A/C was reduced in 7/8 and was undetectable in 4/8 primary GC by immunohistochemistry. However, the change of mRNA level and the clinical significance of this change remain unknown. We thus investigated lamin A/C expression in a large amount of primary GC with RT-PCR, real time RT-PCR, western blot and immunohistochemistry. Additionally, we identified its relationship with clinicopathological features and evaluated its prognostic value to post-resectional survival in GC.

## Methods

### Patients and tissue specimens

A total of 126 primary GC patients treated at the Cancer Center, Sun Yat-sen University from 2001 to 2002 were enrolled to this study, including 88 males and 38 females with a median age of 50 years (range, 21–75 years). All patients were not pretreated with radiotherapy or chemotherapy prior to surgery. With informed consents from each patient, the matching normal (mucosa far and free from tumour invasion, > 5 cm) and tumour tissues were obtained at the time of surgical resection. All tissues were obtained fresh and frozen in liquid nitrogen until process. All specimens were confirmed by pathological examination and staging was performed according to UICC classification (TNM 1997).

### Extraction of total RNA and RT-PCR

Total RNA was extracted from tissues with TRIzol (Invitrogen, Carlsbad, CA) according to the user manual. Levels of lamin A/C mRNA were determined in 52 samples by RT-PCR and 30 samples by real-time RT-PCR with cDNA prepared from total RNA by using a First Strand cDNA Synthesis kit (Roche, Indianapolis, IN).

For RT-PCR reactions, the thermal cycle was defined at 94°C for 5 min, followed by 30 cycles of denaturing at 94°C for 30 s, annealing at 57.5°C for 30 s and extension at 72°C for 30 s, and a final extension at 72°C for 10 min. PCR products were electrophoresed in 1.5% agarose gels and visualized by ethidium bromide staining to check for nonspecific amplification. To quantify the densities of the bands, the gray values were measured with the Bio-Rad imaging system. After the values of lamin A/C were normalized by the corresponding values of β-actin, the ratio of the tumour to the non-tumour gastric tissues was calculated.

For real-time RT-PCR, each reaction was done on a MX3000P real-time PCR instrument with the SYBR PremixEx Taq™ (Takara, Dalian, China) in a 25 μl reaction system with 1 μg cDNA following the manufacturer's protocol. All reactions were repeated three times. β-actin was used as an internal control, and measurements between samples were compared by the threshold cycle of amplification (C_T_). The fold change in expression levels was determined by a comparative C_T _method using the formula: ^ΔΔCT^(ΔΔC_T _= (C_T (lamin A/C) _- C_T (β-action) _)_cancer _- (C_T (lamin A/C) _- C_T (β-action)_)_normal_). Primer sequences used for lamin A/C are: forward 5'-CGGTTCCCACCAAAGTTCA-3' and reverse 5'-CTCATCCTCGTCGTCCTCAA-3'; for β-actin: forward 5'-CACCCAGCACAATGAAGAT-3' and reverse 5'-CAAATAAAGCCATGCCAAT-3'. The primers were designed between different exons and encompassing large introns to avoid any amplification of genomic DNA. QPCR was performed for pre-denaturing at 95 °C for 10 seconds, followed by 40 cycles (95°C for 5 seconds and 57.5°C for 20 seconds).

### Western-blot analysis

Western blot was performed on 34 tumour specimens and corresponding adjacent non-cancerous samples. The frozen tissues were lysed in RIPA buffer plus protease inhibitors PMSF (Sangon, Shanghai, China), and the resulting insoluble material removed by centrifugation at 12,000 g 4°C for 30 min. After concentration measured by the BCA method, protein samples were electrophoresed on 12% sodium dodecyl sulphate (SDS)-polyacrylamide gels and subsequently transferred to a PVDF membrane (Millipore, Billerica, MA) by electroblotting. After blocking for 1 h in Tris buffered saline (pH 7.6, containing 0.1% Tween and 5% non-fat milk) at room temperature, membranes were incubated overnight at 4°C with primary polyclonal antibody against lamin A/C (Cell Signaling, Danvers, MA, at 1:1000 dilution), and β-actin (Abcam, Cambridge, UK, at 1:2000 dilution) with gentle shaking. After washing, the membrane was then probed with the appropriate secondary antibody for 60 min at room temperature. Protein binding on the membrane was detected by the enhanced chemiluminescence (ECL) detection system (Pierce, Rockford, IL) according to the manufacturer's instructions. Then band intensity was measured by densitometry using the Quantity One software (Bio-Rad, Hercules, CA). The protein levels were normalized with respect to β-actin protein level.

### Immunohistochemistry analysis

Sections (4 μm thick) of formalin fixed, paraffin wax blocks were cut onto polylysine-coated microscope slides. According to the specification of IHC S-P detection kit (Maixin, Fujian, China): after deparaffinisation in xylene and hydration through graded alcohol, sections were washed and then exposed to microwave pretreatment (in 10 mM citrate buffer, pH 6 at 850W for two periods of five minutes) to enhance antigenicity. Endogenous peroxidase was blocked with 3% hydrogen peroxide for 10 min and non-specific binding was blocked with 5% normal goat serum in phosphate buffered saline for 15 min. Then sections were incubated with first antibody (rabbit-anti-human lamin A/C protein polyclonal antibody, Cell Signaling, Danvers, MA) at a concentration of 1: 200 at 4°C overnight. Biotinylated antirabbit IgG antibody (Boshide, Wuhan, China) was added for 15 min at 37°C, following the incubation with streptavidin-biotin/horseradish peroxidase complex for 10 min at 37°C. Finally, sections were colored with 3,3'-diaminobenzidine tetrahydrochloride (DAB) for 5 min, lightly counterstained with hematoxylin and mounted. Sections immunostained with PBS replacing primary antibody are used as negative control. A positive control was included with each batch of staining to ensure consistency between consecutive runs. The brown-yellow staining of nuclear membrane was considered positive. For each case, the entire stained tissue section was scanned, choosed 5 visual fields at 400× magnification randomly and count 100 cells each field. The degree of immunointensity was quantified by using the total immunostaining score calculated as the sum of the positive percentage of stained tumour cells and the staining intensity. The positive percentage was scored as '0' (< 5%, negative), '1' (5–25%, sporadic), '2' (25–50%, focal), '3' (> 50%, diffuse). The staining intensity was score as '0' (no staining), '1' (weakly stained), '2' (moderately stained), and '3' (strongly stained). Cases with weighted scores of less than 1 were defined as negative; otherwise they were defined as positive. No folding, and edging-effect fields were chosen during calculation of 100 cells per five fields. The score assessment was performed independently by two pathologists.

### Statistical analysis

Quantitative values were expressed as means ± SD. Comparison of the mRNA and protein expression level of lamin A/C between tumour and control was made with Paired-samples *t*-test in all cases. Categorical variables were enumeration data of counting the number of samples. The correlation of lamin A/C expression with various clinicopathological parameters was calculated with Chi-square test for proportion and Pearson's regression analysis. Overall survival was measured from the time of surgery until death with disease, or until the end of follow up. Patients who died of causes unrelated to the disease were censored at the time of death. Survival curves were calculated by the Kaplan-Meier method, and the differences between the curves were analyzed with the log-rank test. Cox proportional-hazard analysis was used for univariate and multivariate analysis to explore the effect of clinicopathological variables and the Lamin A/C expression on survival. All statistical analyses were performed in the SPSS 15.0 software and a *P *value < 0.05 was considered statistically significant.

## Results

### RT-PCR and real time RT-PCR analysis

**Figure 1:**
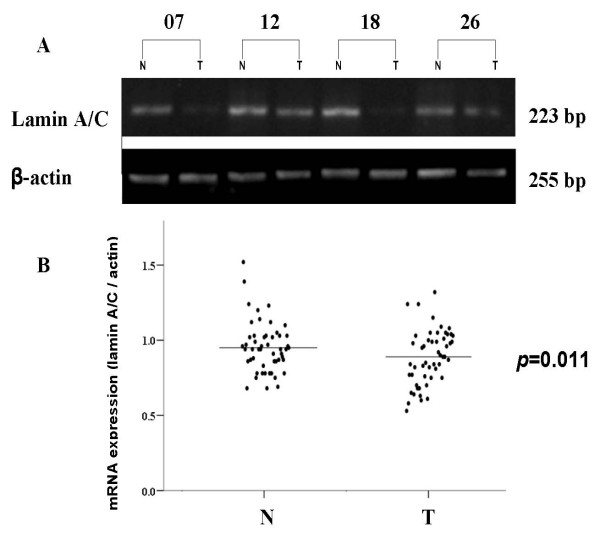
**Expression pattern of lamin A/C in GC specimens by RT-PCR**. (A) 1.5% agarose electrophoresis of lamin A/C products of RT-PCR in GC specimens. Representative results from 4 pairs of GC and corresponding normal gastric tissues are shown. β-actin was used as an internal quantitative control. (B) Densitometry analyses of lamin A/C mRNA level quantified by compared with β-actin in GC and corresponding normal gastric samples. The expression of lamin A/C gene was reduced in tumour tissues when compared with corresponding non-tumourous tissues (*p *= 0.011). T, GC; N, corresponding non-cancerous tissues.

The expression levels of lamin A/C mRNA were examined in 52 paired clinical samples by semiquantitative RT-PCR. As shown in Fig. 1A, lamin A/C mRNA could be detected in GC tissues as well as in matched non-cancerous tissues. However, a large decrease in the levels of lamin A/C mRNA expression was observed in primary GC as compared with normal tissue. The analysis of results displayed the density value (normalized to β-actin expression as a loading control) of tumour was significantly lower than that in corresponding non-cancerous tissue using paired t-test (*p *= 0.011, Fig. 1B).

**Figure 2:**
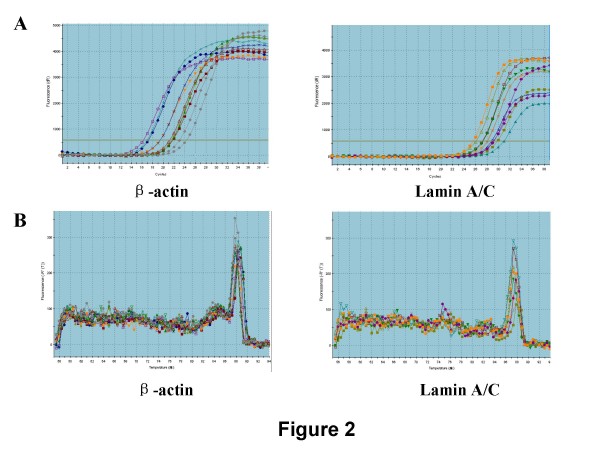
**The dissociation curves and amplification curves of lamin A/C in GC specimens by real time RT-PCR**. Representative results from 5 pairs of GC and corresponding normal gastric tissues are shown. (A) The dissociation curves of lamin A/C and β-actin. (B) The amplification curves of lamin A/C and β-actin.

To validate the results of semiquantitative RT-PCR, we randomly selected 30 cases out of the 52 patients to investigate the mRNA expression level with real time RT-PCR. The dissociation curve and amplification curve were shown in Fig. 2A and 2B. The fold change in expression levels determined by a comparative C_T _method also demonstrated that lamin A/C expression is reduced in GC tissues. We further analyzed the correlations between lamin A/C mRNA expression and clinicopathological features. As shown in Table 1, the mRNA expression level was evidently lower in poor differentiated tumours than that in well or moderately differentiated tumours. Decreased of lamin A/C expression correlated with histological differentiation significantly (r = 0.438, *p *= 0.025). However, there were no statistical correlations between lamin A/C and invasion, tumour size and metastasis.

**Table 1:** Correlations between lamin A/C expression detected by real time RT-PCR and pathological variables in 30 cases of GC

Variables	Number of Cases	Fold Change (mean ± SD)	t	*p*-Value
**Invasion**				
Profound layer	24	0.77 ± 0.19	-0.692	0.495
Superficial layer	6	0.83 ± 0.19		
**Differentiation**				
Poor	21	0.73 ± 0.19	-2.376	0.025^a^
Well or Moderate	9	0.90 ± 0.13		
**Metastasis**				
No	23	0.76 ± 0.18	-0.792	0.435
Yes	7	0.83 ± 0.23		
**Tumour Size (cm)**				
< 5	18	0.83 ± 0.18	1.704	0.099
≥5	12	0.71 ± 0.20		

^a ^Statistically significant (p < 0.05).

### Western blot analysis

**Figure 3:**
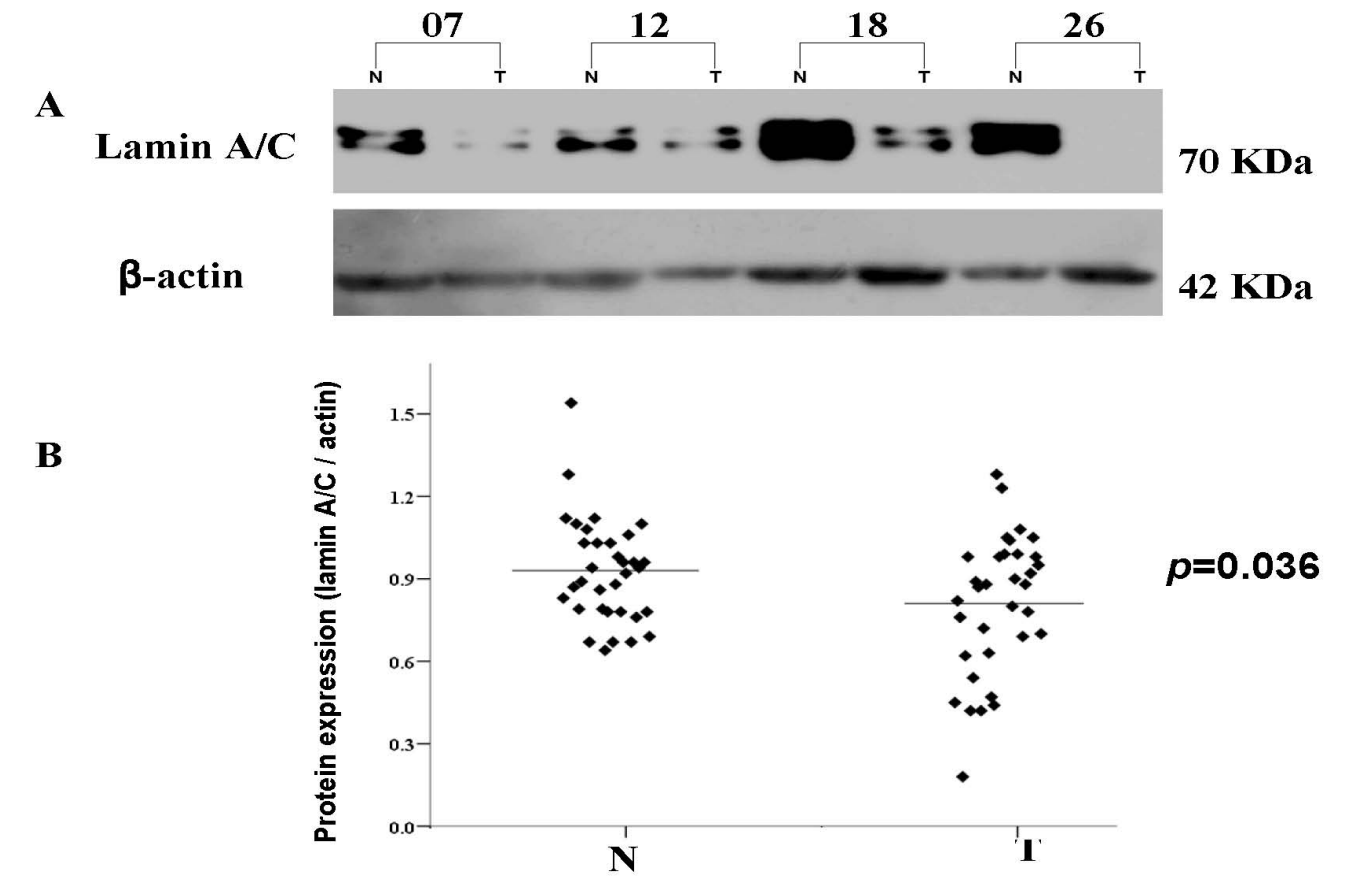
**Expression pattern of lamin A/C in GC specimens by Western Blot**. (A) Representative results from 4 pairs of GC and corresponding normal gastric tissues are shown. β-actin was used as an internal quantitative control. (B) Densitometry analyses of lamin A/C protein level quantified by compared with β-actin in GC and corresponding normal gastric samples. The expression of lamin A/C gene was reduced in tumour tissues when compared with corresponding non-tumourous tissues (*p *= 0.036). T, GC; N, corresponding non-cancerous tissues.

Western blot was performed on 34 tumour specimens and corresponding adjacent non-cancerous samples to further investigate if the expression of lamin A/C is reduced at protein levels. Western blot showed a lamin A/C band at the expected 70 kDa size and the amount of lamin A/C protein was measured by densitometry. Lamin A/C protein expression was decreased in 47% (16/34) of gastric cancer tissues in comparison with the adjacent normal tissues, as shown in Figure 3A. The 16 cases of reduced lamin A/C protein level of cancerous gastric tissues compared with the normal matched tissues included 13 cases with reduced expression on mRNA level and 3 cases even without the transcriptional reduction. The analysis of results displayed that the density value (normalized to β-actin expression as a loading control) of tumour was significantly lower than that of corresponding noncancerous tissue (*P *= 0.036) (Fig. 3B). These data are in agreement with the results from the RT-PCR analysis for lamin A/C expression in patients with gastric cancer.

### Immunohistochemistry analysis

**Figure 4:**
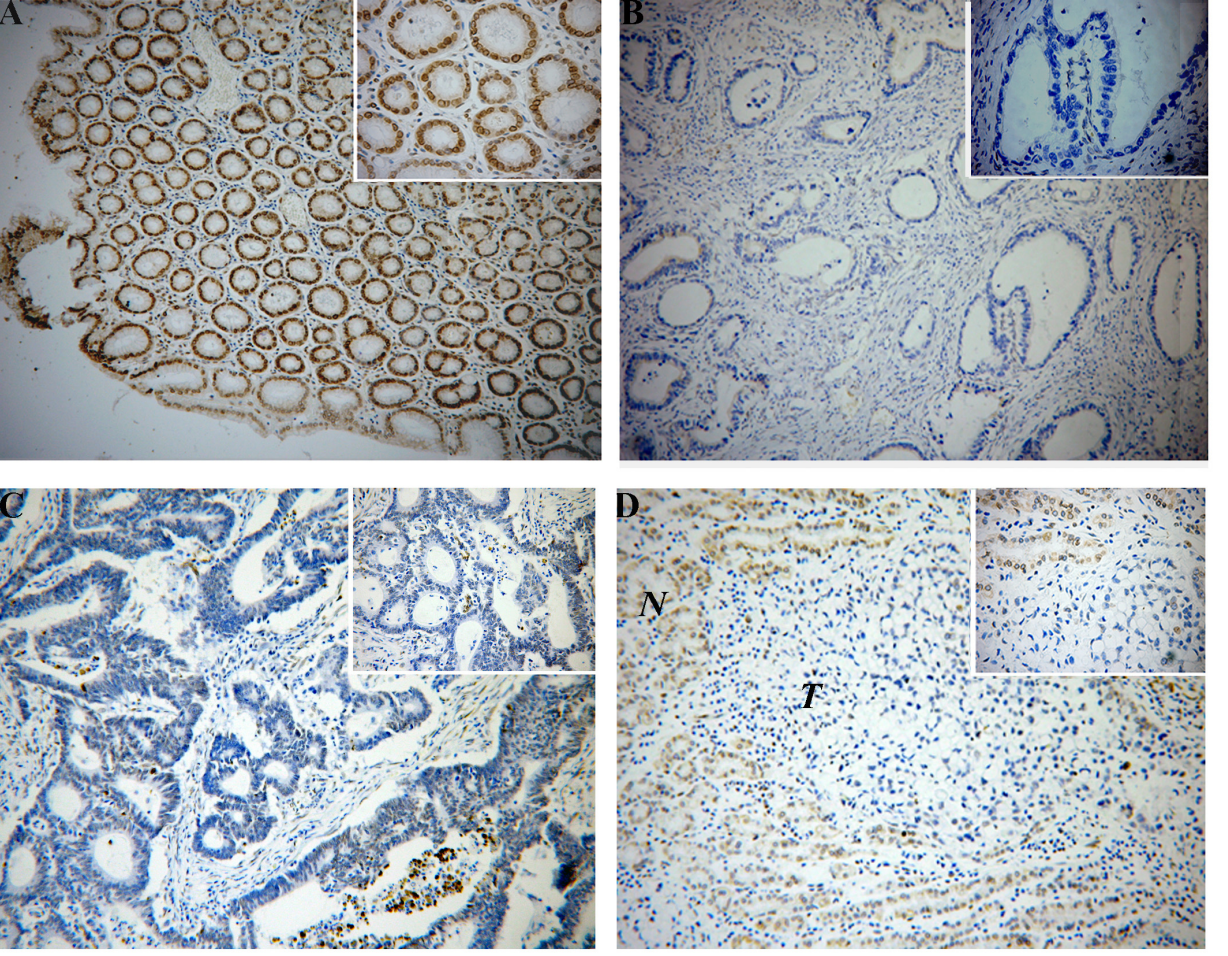
**Immunohistochemical detection of Lamin A/C protein expression in GC and surrouding non-cancerous tissues**. Positive staining was mostly seen on nuclear of epithelial cells. (A) positive staining of Lamin A/C in normal gastric mucosa(× 100). (B) negative staining of Lamin A/C in well-differentiated gastric carcinoma(× 100). (C) negative staining of Lamin A/C in moderately differentiated gastric carcinoma(× 100). (D) negative staining of Lamin A/C in gastric signet-ring cell carcinoma(× 100). T, GC; N, corresponding non-cancerous tissues. The right upper frame of each figure showing high-power field(× 400).

Lamin A/C immunostaining were strong brown-yellow in 96% (121/126) normal gastric mucosal epithelial cells, with location to nuclear membrane, while only 4% (5/126) samples were negative(Figure 4A). However, in tumour tissues, the positive rate of lamin A/C protein expression was only 55.6% (70/126), while negative rate was 44.4% (56/126) (Fig. 4B, C and 4D). We often observed a sharp contrast between infiltrative tumour areas of negative staining and the adjacent tissue of positive staining (Fig. 4D). Compared with normal tissues, there is evident weaken of lamin A/C immunoreactivity in GC samples with significant difference (*p *= 0.016). We also did an analysis concerning the correlation between the expression of lamin A/C and the clinicopathological variables. As shown in Table 1, the positive rate of lamin A/C expression was 78.9%, 65.1%, 51.6% and 35% in well-differentiated, moderately-differentiated, poorly-differentiated adenocarcinoma and undifferentiated carcinoma, respectively. There was a significant difference between histological type and expression of lamin A/C, the lower the differentiation, the more the absence of lamin A/C presence(r = 0.361, *p *= 0.034). There was no apparent relevance between lamin A/C protein expression and patient gender, age, tumour size, distant metastasis, lymph node involvement, the depth of invasion or clinical staging (*P *> 0.05 respectively)(Table 2).

**Table 2:** Association of Lamin A/C immunostaining with clinicopathological parameters in 126 cases of primary GC

Clinicopathological variable	Cases (n = 126)	Lamin A/C		*p*-value
			
		positive (%)	negative (%)	
			
		n = 70	n = 56	
**Gender**				0.410
male	88	51 (58.0)	37 (42.0)	
female	38	19 (50.0)	19 (50.0)	
**Age (years)^a^**				0.905
< 56	60	33 (55.0)	27 (45.0)	
≥ 56	66	37 (56.1)	29 (43.9)	
**Tumour size (cm)^a^**				0.902
< 5	78	43 (55.1)	35 (44.9)	
≥ 5	48	27 (56.3)	21 (43.7)	
**Depth of invasion**				0.870
T1	9	6 (66.7)	3 (33.3)	
T2	22	12 (54.5)	10 (45.5)	
T3	75	42(56.0)	33 (44.0)	
T4	20	10 (50.0)	10 (50.0)	
**Lymph node metastasis^b^**				0.550
N_0_	42	23 (54.8)	19 (45.2)	
N_1_	36	22 (61.1)	14 (38.9)	
N_2_	38	18 (47.4)	20(52.6)	
N_3_	10	7(70.0)	3 (30.0)	
**Distant metastasis**				0.659
M0	101	55 (54.5)	40 (45.5)	
M1	25	15(60.0)	10 (40.0)	
**Staging**				0.894
I	17	10 (58.8)	7 (41.2)	
II	27	14 (51.9)	13 (48.1)	
III	47	25 (53.2)	22 (46.8)	
IV	35	21 (60.0)	14 (40.0)	
**Differentiation**				0.034^c^
well	19	15(78.9)	4 (21.1)	
moderate	20	13(65.0)	7 (35.0)	
poor	67	35(51.6)	32 (48.4)	
undifferentiated	20	7 (35.0)	13 (65.0)	

^a^grouping of age and tumour size was performed according to median.

^b ^grouping of staging and lymph node metastasis was performed according to UICC classification (TNM 1997).

^c^statistical significance (*p *< 0.05)

### Correlation between lamin A/C expression and patients' survival

**Figure 5:**
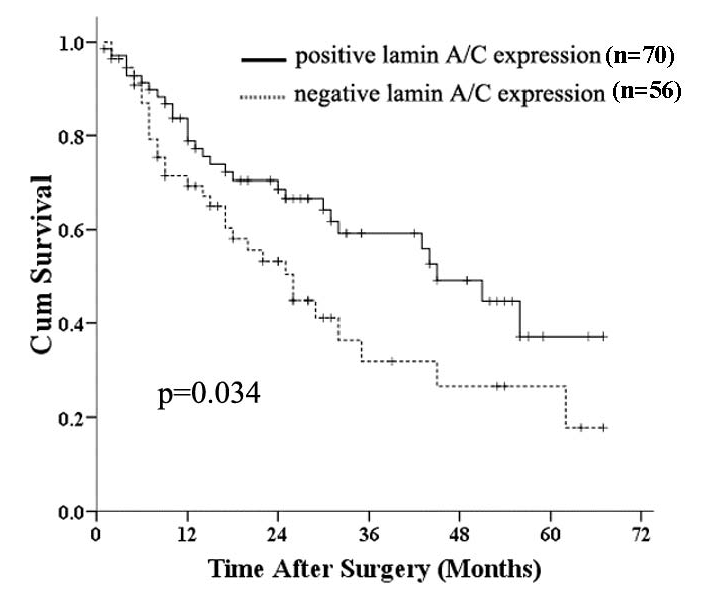
**Estimated overall survival according to the expression of lamin A/C in 126 cases of GCs (the Kaplan – Meier method)**. Based on the results of immunohistochemical staining, the expression of lamin A/C was classified as the negative expression (n = 56) and the positive (n = 70). Log-rank test shows that GC patients with the negative lamin A/C expression showed significantly poorer prognosis than those with the positive expression.

Using Kaplan-Meier curve method, we evaluated the relationship between the lamin A/C expression and the outcome of 126 patients. The overall survival rates were 58.6% and 44.6%, respectively, in patients with positive and negative lamin A/C expression. Of 70 lamin A/C immunohistochemical positive-staining patients, the median survival time is 45.0 ± 5.5 months, while that of 56 negative-staining patients is 26.0 ± 4.2 months. There was a significantly longer median survival time in the lamin A/C protein-positive group than in the negative group (*P *= 0.034, log-rank test; Fig. 5). Univariate Cox regression analysis also identified that clinical variables including tumour invasion and metastasis were significantly associated with overall survival (Table 3). Furthermore, to evaluate the potential of negative lamin A/C expression (negative vs. positive) as an independent predictor for overall survival of GC, multivariate Cox regression analyses were performed. While tumour invasion failed to demonstrate independency, only status of metastasis and negative lamin A/C expression may play a role to predict the overall survival in GC (*p *= 0.040 and *p *= 0.041, respectively; Table 3).

**Table 3:** The overall survival univariate and multivariate Cox regression analysis

Clinicopathological Variable	Relative Risk (95% CI)	*p*-Value
*Univariate*		
Gender	0.948 (0.549–1.637)	0.038
Tumour Size	1.621 (0.974–2.697)	0.063
Metastasis	2.057 (1.110–3.810)	0.022 ^a^
Invasion	2.012 (1.098–3.698)	0.024^a^
Stage	0.915 (0.709–1.181)	0.497
Histological Differentiation	1.704 (0.969–2.997)	0.064
Lamin A/C	0.582 (0.349–0.969)	0.038^a^
		
*Multivariate*		
Metastasis	1.905 (1.029–3.526)	0.040^a^
Lamin A/C	0.585 (0.350–0.978)	0.041^a^

Abbreviation: 95% CI, 95% confidence interval.

^a ^Statistically significant (p < 0.05).

## Discussion

A-type lamins are essential components of the nuclear lamina [8]. Aside from their structural role in the formation of the nuclear lamina, lamins A and C are found in the nucleoplasm adjacent to sites of DNA synthesis and RNA processing, suggesting that these proteins could influence both DNA replication and gene expression [2-4]. The A-type lamins, lamins A and C, are synthesized from alternatively spliced transcripts of lamin A gene (LMNA) [9,10]. A-type lamins are absent in early embryonic development and in certain stem cell populations in adults [11-13] and are expressed only after commitment of cells to a particular differentiation pathway [12,14]. Mutations in LMNA produce an intriguingly diverse spectrum of diseases including muscular dystrophies (Emery-Dreifuss muscular dystrophy, limb-girdle muscular dystrophy type 1B), neuropathy (Charcot-Marie-Tooth disease type 2), dilated cardiomyopathy with conduction system disease, familial partial lipodystrophy (s.c. fat loss and diabetes), mandibuloacral dysplasia (skeletal malformations and lipodystrophy), atypical Werner's syndrome, and Hutchinson-Gilford progeria syndrome(precocious aging syndromes) [15-19]. To date, some 200 mutations have been identified in LMNA.

Numerous studies have shown that reduced or absent lamin A/C expression is a common feature of a variety of different cancers, notwithstanding there is little direct evidence connecting tumourigenesis to A-type lamins [5,7,20-23]. Besides maintain the normal nuclear structure, the lamins and lamin-associated proteins are also required for most other nuclear activities including DNA replication, RNA Pol II-dependent transcription, migration and anchorage of nuclei, correct spacing of nuclear pore complexes, regulation of mitosis, and apoptosis [3]. With respect to its multiple functions, it is convincible to presume that change of lamin A/C protein may contribute to tumourigenesis and progression.

The development of GC is a multistep process and phenotypic changes during cancer progression reflect the sequential accumulation of genetic alterations in cells. Carcinogenesis and progression of human GC are related to the activation of proto-oncogenes and/or the inactivation of tumour suppressor genes. Moss et al [7] detected the expression of lamin A/C in 8 primary GC patients by immunohistochemistry, they found reduced expression of lamin A/C in 7/8 patients. The case number studied in that report was relatively small, and the change of mRNA level and the clinical significance of this change were not investigated. We did this study on over one hundred cases of primary GC to elucidate the expression change of lamin A/C and its clinicopathological correlation. This study clearly showed that lamin A/C mRNA as well as protein was down-regulated in GC tissues compared with the adjacent normal tissues, suggesting that lower expression of lamin A/C occurred not only at the post-transcriptional level, but also at the transcriptional level in GC samples. In addition, correlation analysis based on real time RT-PCR revealed that lamin A/C mRNA expression is associated with histological differentiation in GC.

Furthermore, we examined the expression of lamin A/C in primary gastric cancer and their relationships with clinicopathological characteristics. Compared with only 4% (5/126) negative staining in normal gastric samples, there was a higher negative rate of 44.4% (56/126) in tumour tissues. Compared with normal tissues, there is evident weaken of lamin A/C immunoreactivity in GC samples with significant difference (*p *= 0.016). In addition, statistical analysis demonstrated an evident correlation between expression of lamin A/C and histological type. With the progression of tumour, the percentage of negative lamin A/C expression was also growing, which is consistent with previous conclusion that lamin A/C is expressed only in later stages of development and in differentiated cells. The low expression of lamin A/C mRNA and protein observed in gastric carcinoma suggests that loss of lamin A/C involves in the development of human gastric carcinoma.

A number of groups have reported that A-type lamins, in contrast to B-type lamins, are differentially expressed in embryonic tissues [12,13,24]. Undifferentiated cells or cells at early stages of differentiation were found to lack A-type lamin expression. In addition, A-type lamins were not expressed in proliferating cells of some adult human tissues such as the basal keratinocytes of the skin[25]. This could probably explain why more poorly differentiated gastric tumour tissues lack lamin A/C expression.

Another important discovery in our study was that decreased expressions of lamin A/C was significantly correlated with poor patient outcome. Patients with gastric cancer who were lamin A/C protein-negative had a worse 5-year survival rate. Although there has been a great improvement in the diagnosis and treatment with gastric cancer recently, it is still a major health problem and a leading cause of cancer mortality in Asian countries. To identify reliable prognostic markers in gastric cancer is therefore very important to guide surgical and chemotherapeutic treatment according to individual risk. This finding suggested that lamin A/C may have diagnostic and therapeutic potential for patients with gastric cancer in order to design optimal individual treatment modalities.

The mechanism of tumour suppression by lamin A/C is not fully understood. Biochemical studies have shown that lamin A/C can interact with different gene regulators including SREBP1, MOK2 and the retinoblastoma protein (pRB) [26-28]. Excitingly, a series of experiments demonstrated that lamin A/C is necessary for a generally known tumour suppressor – pRB stabilization, and decreased expression of lamin A/C results in reduced activity of pRB [29-31]. pRB is a critical regulator of cell proliferation and differentiation and an important tumor suppressor. In the G(1) phase of the cell cycle, pRB localizes to perinucleolar sites associated with lamin A/C intranuclear foci. Johnson et al[32] examined pRB function in cells lacking lamin A/C, finding that pRB levels are evidently decreased and that the remaining pRB is mislocalized. They demonstrated that A-type lamins protect pRB from proteasomal degradation. Both pRB levels and localization are restored upon reintroduction of lamin A. Lmna(-/-) cells resemble Rb(-/-) cells, exhibiting altered cell-cycle properties and reduced capacity to undergo cell-cycle arrest in response to DNA damage. Their findings established a functional link between a core nuclear structural component and an important cell-cycle regulator. Recently, there was another report showing that protein levels of the oncoprotein gankyrin are elevated in Lmna^-/- ^fibroblasts and Lmna^-/- ^cells are refractory to p14^arf^-mediated cell cycle arrest, as was previously shown with p16^ink4a ^[33]. These findings together with our data increase the possibility that lamin A/C might function as a tumour suppressor through function as a negative regulator of cell growth.

However, the molecular mechanism underlying the loss of lamin A/C in human cancer remains unknown. Transcriptional inactivation by CpG island promoter hypermethylation is a well-established mechanism for gene silencing in human tumours and may represent a frequent mechanism by which the gene is inactivated during tumourigenesis [34]. Methylation of the promoter region is an alternative mechanism to intragenic mutations for the inactivation of tumour suppressor genes and plays an important role in tumourigenesis [35]. Classical tumour suppressor genes and genes involved in chemosensitivity, such as hMLH1, p16, p15, Rb, VHL, E-cadherin, GSTP1, and BRCA1, or the DNA repair gene MGMT, undergo epigenetic inactivation by hypermethylation of their regulatory regions [36-39]. Researchers demonstrated the presence of promoter CpG island hypermethylation in lamin A/C gene and correlated this to loss of mRNA and protein expression in leukemia and lymphoma malignancies [40]. Furthermore, they also reported that lamin A/C CpG island promoter hypermethylation is a significant predictor of shorter failure-free survival and overall survival in nodal diffuse large B-cell lymphomas. This epigenetic alteration could explain why somatic mutation of lamin A/C was not detected in cancer cells.

## Conclusion

We found a significant lower lamin A/C expression level in gastric cancer tissues compared with non-cancerous gastric tissues, and loss of lamin A/C expression correlates with histological classification. Our results suggest lamin A/C may play a suppressive role in tumourigenesis of gastric cancer. Lamin A/C could serve as a useful prognostic marker in primary gastric cancer patients and a therapeutic target to prevent gastric carcinoma. However, to elucidate the molecular mechanisms of lamin A/C in gastric carcinogenesis, further studies are still needed to be done.

## Competing interests

The authors declare that they have no competing interests.

## Authors' contributions

ZRW designed the research and wrote the paper. ZRW and DSW carried out the molecular genetics studies and data analysis. DSW and XD collected the gastric cancer tissues. ZRW and JG carried out the pathological diagnosis. FZ and LRW prepared the tissue slides. All authors have read and approved the manuscript.

## References

[B1] Stewart CL, Kozlov S, Fong LG, Young SG (2007). Mouse models of the laminopathies. Exp Cell Res.

[B2] Zink D, Fischer AH, Nickerson JA (2004). Nuclear structure in cancer cells. Nat Rev Cancer.

[B3] Ostlund C, Worman HJ (2003). Nuclear envelope proteins and neuromuscular diseases. Muscle Nerve.

[B4] Worman HJ, Courvalin JC (2004). How do mutations in lamins A and C cause disease?. J Clin Invest.

[B5] Prokocimer M, Margalit A, Gruenbaum Y (2006). The nuclear lamina and its proposed roles in tumorigenesis: projection on the hematologic malignancies and future targeted therapy. J Struct Biol.

[B6] Jemal A, Siegel R, Ward E, Murray T, Xu J, Thun MJ (2007). Cancer statistics, 2007. CA Cancer J Clin.

[B7] Moss SF, Krivosheyev V, de Souza A, Chin K, Gaetz HP, Chaudhary N, Worman HJ, Holt PR (1999). Decreased and aberrant nuclear lamin expression in gastrointestinal tract neoplasms. Gut.

[B8] Lin F, Worman HJ (1993). Structural organization of the human gene encoding nuclear lamin A and nuclear lamin C. J Biol Chem.

[B9] Fisher DZ, Chaudhary N, Blobel G (1986). cDNA sequencing of nuclear lamins A and C reveals primary and secondary structural homology to intermediate filament proteins. Proc Natl Acad Sci USA.

[B10] Sinensky M, Fantle K, Trujillo M, McLain T, Kupfer A, Dalton M (1994). The processing pathway of prelamin A. J Cell Sci.

[B11] Burke B, Stewart CL (2002). Life at the edge: the nuclear envelope and human disease. Nat Rev Mol Cell Biol.

[B12] Rober RA, Weber K, Osborn M (1989). Differential timing of nuclear lamin A/C expression in the various organs of the mouse embryo and the young animal: a developmental study. Development.

[B13] Stewart C, Burke B (1987). Teratocarcinoma stem cells and early mouse embryos contain only a single major lamin polypeptide closely resembling lamin B. Cell.

[B14] Oguchi M, Sagara J, Matsumoto K, Saida T, Taniguchi S (2002). Expression of lamins depends on epidermal differentiation and transformation. Br J Dermatol.

[B15] Brodsky GL, Muntoni F, Miocic S, Sinagra G, Sewry C, Mestroni L (2000). Lamin A/C gene mutation associated with dilated cardiomyopathy with variable skeletal muscle involvement. Circulation.

[B16] Csoka AB, Cao H, Sammak PJ, Constantinescu D, Schatten GP, Hegele RA (2004). Novel lamin A/C gene (LMNA) mutations in atypical progeroid syndromes. J Med Genet.

[B17] De Sandre-Giovannoli A, Bernard R, Cau P, Navarro C, Amiel J, Boccaccio I, Lyonnet S, Stewart CL, Munnich A, Le Merrer M, Levy N (2003). Lamin a truncation in Hutchinson-Gilford progeria. Science.

[B18] Hegele RA, Cao H, Anderson CM, Hramiak IM (2000). Heterogeneity of nuclear lamin A mutations in Dunnigan-type familial partial lipodystrophy. J Clin Endocrinol Metab.

[B19] Vantyghem MC, Pigny P, Maurage CA, Rouaix-Emery N, Stojkovic T, Cuisset JM, Millaire A, Lascols O, Vermersch P, Wemeau JL, Capeau J, Vigouroux C (2004). Patients with familial partial lipodystrophy of the Dunnigan type due to a LMNA R482W mutation show muscular and cardiac abnormalities. J Clin Endocrinol Metab.

[B20] Broers JL, Raymond Y, Rot MK, Kuijpers H, Wagenaar SS, Ramaekers FC (1993). Nuclear A-type lamins are differentially expressed in human lung cancer subtypes. Am J Pathol.

[B21] Jansen MP, Machiels BM, Hopman AH, Broers JL, Bot FJ, Arends JW, Ramaekers FC, Schouten HC (1997). Comparison of A and B-type lamin expression in reactive lymph nodes and nodular sclerosing Hodgkin's disease. Histopathology.

[B22] Stadelmann B, Khandjian E, Hirt A, Luthy A, Weil R, Wagner HP (1990). Repression of nuclear lamin A and C gene expression in human acute lymphoblastic leukemia and non-Hodgkin's lymphoma cells. Leuk Res.

[B23] Venables RS, McLean S, Luny D, Moteleb E, Morley S, Quinlan RA, Lane EB, Hutchison CJ (2001). Expression of individual lamins in basal cell carcinomas of the skin. Br J Cancer.

[B24] Paulin-Levasseur M, Scherbarth A, Traub U, Traub P (1988). Lack of lamins A and C in mammalian hemopoietic cell lines devoid of intermediate filament proteins. Eur J Cell Biol.

[B25] Chaudhary N, Cance WG, Worman HJ, Blobel G, Cordon-Cardo C (1990). Nuclear lamin expression in normal and neoplastic human tissues. J Cell Biol.

[B26] Ozaki T, Saijo M, Murakami K, Enomoto H, Taya Y, Sakiyama S (1994). Complex formation between lamin A and the retinoblastoma gene product: identification of the domain on lamin A required for its interaction. Oncogene.

[B27] Dreuillet C, Tillit J, Kress M, Ernoult-Lange M (2002). In vivo and in vitro interaction between human transcription factor MOK2 and nuclear lamin A/C. Nucleic Acids Res.

[B28] Lloyd DJ, Trembath RC, Shackleton S (2002). A novel interaction between lamin A and SREBP1: implications for partial lipodystrophy and other laminopathies. Hum Mol Genet.

[B29] Johnson BR, Nitta RT, Frock RL, Mounkes L, Barbie DA, Stewart CL, Harlow E, Kennedy BK (2004). A-type lamins regulate retinoblastoma protein function by promoting subnuclear localization and preventing proteasomal degradation. Proceedings of the National Academy of Sciences of the United States of America.

[B30] Nitta RT, Jameson SA, Kudlow BA, Conlan LA, Kennedy BK (2006). Stabilization of the retinoblastoma protein by A-type nuclear lamins is required for INK4A-mediated cell cycle arrest. Molecular and Cellular Biology.

[B31] Pekovic V, Harborth J, Broers JL, Ramaekers FC, van Engelen B, Lammens M, von Zglinicki T, Foisner R, Hutchison C, Markiewicz E (2007). Nucleoplasmic LAP2alpha-lamin A complexes are required to maintain a proliferative state in human fibroblasts. J Cell Biol.

[B32] Johnson BR, Nitta RT, Frock RL, Mounkes L, Barbie DA, Stewart CL, Harlow E, Kennedy BK (2004). A-type lamins regulate retinoblastoma protein function by promoting subnuclear localization and preventing proteasomal degradation. Proc Natl Acad Sci USA.

[B33] Nitta RT, Smith CL, Kennedy BK (2007). Evidence that proteasome-dependent degradation of the retinoblastoma protein in cells lacking A-type lamins occurs independently of gankyrin and MDM2. PLoS ONE.

[B34] Plass C (2002). Cancer epigenomics. Hum Mol Genet.

[B35] Sugimura T, Ushijima T (2000). Genetic and epigenetic alterations in carcinogenesis. Mutat Res.

[B36] Ogi K, Toyota M, Ohe-Toyota M, Tanaka N, Noguchi M, Sonoda T, Kohama G, Tokino T (2002). Aberrant methylation of multiple genes and clinicopathological features in oral squamous cell carcinoma. Clin Cancer Res.

[B37] Kang GH, Shim YH, Jung HY, Kim WH, Ro JY, Rhyu MG (2001). CpG island methylation in premalignant stages of gastric carcinoma. Cancer Res.

[B38] Ding Y, Le XP, Zhang QX, Du P (2003). Methylation and mutation analysis of p16 gene in gastric cancer. World J Gastroenterol.

[B39] Jung HY, Jung KC, Shim YH, Ro JY, Kang GH (2001). Methylation of the hMLH1 promoter in multiple gastric carcinomas with microsatellite instability. Pathol Int.

[B40] Agrelo R, Setien F, Espada J, Artiga MJ, Rodriguez M, Perez-Rosado A, Sanchez-Aguilera A, Fraga MF, Piris MA, Esteller M (2005). Inactivation of the lamin A/C gene by CpG island promoter hypermethylation in hematologic malignancies, and its association with poor survival in nodal diffuse large B-cell lymphoma. J Clin Oncol.

